# A mixed-methods study of challenges experienced by clinical teams in measuring improvement

**DOI:** 10.1136/bmjqs-2018-009048

**Published:** 2019-08-24

**Authors:** Thomas Woodcock, Elisa G Liberati, Mary Dixon-Woods

**Affiliations:** 1 CLAHRC for Northwest London, Imperial College, Chelsea and Westminster Hospital Campus, London, UK; 2 THIS Institute (The Healthcare Improvement Studies Institute), Department of Public Health and Primary Care, University of Cambridge, Cambridge, UK

**Keywords:** quality improvement, quality measurement, evaluation methodology

## Abstract

**Objective:**

Measurement is an indispensable element of most quality improvement (QI) projects, but it is undertaken to variable standards. We aimed to characterise challenges faced by clinical teams in undertaking measurement in the context of a safety QI programme that encouraged local selection of measures.

**Methods:**

Drawing on an independent evaluation of a multisite improvement programme (Safer Clinical Systems), we combined a qualitative study of participating teams’ experiences and perceptions of measurement with expert review of measurement plans and analysis of data collected for the programme. Multidisciplinary teams of frontline clinicians at nine UK NHS sites took part across the two phases of the programme between 2011 and 2016.

**Results:**

Developing and implementing a measurement plan against which to assess their improvement goals was an arduous task for participating sites. The operational definitions of the measures that they selected were often imprecise or missed important details. Some measures used by the teams were not logically linked to the improvement actions they implemented. Regardless of the specific type of data used (routinely collected or selected ex novo), the burdensome nature of data collection was underestimated. Problems also emerged in identifying and using suitable analytical approaches.

**Conclusion:**

Measurement is a highly technical task requiring a degree of expertise. Simply leveraging individual clinicians’ motivation is unlikely to defeat the persistent difficulties experienced by clinical teams when attempting to measure their improvement efforts. We suggest that more structural initiatives and broader capability-building programmes should be pursued by the professional community. Improving access to, and ability to use repositories of validated measures, and increasing transparency in reporting measurement attempts, is likely to be helpful.

## Introduction

Measurement is essential to improving quality and safety in healthcare processes and outcomes.^1 2^ Yet the available evidence suggests that many quality improvement projects may fail to generate reliable or useful data because of challenges in measurement, data collection and interpretation.^3–6^ Characteristic problems include missing data or insufficient data points; insufficient baseline periods; poorly chosen, unclear or changing sampling strategies; poorly annotated data; failure to verify data entry; and poorly chosen or executed analytic strategies.^7 8^ Benn and colleagues^9^ found many of these problems when teams sought to implement data collection and analysis systems in local settings as part of a large-scale quality improvement programme. Similarly, a study of a national system for surveillance of healthcare-associated infections^10^ found variability in how well intensive care units designed their data collection systems and in how they interpreted data.

Failure to produce reliable data about improvement and to interpret it correctly is an important challenge for quality improvement, limiting the inferences that can be made about the success or otherwise of improvement interventions, as well as eroding confidence in the evidence base for improvement.^11^ This problem manifests in summative evaluations and also affects programmes while they are running, when data have potential to be used formatively to optimise the improvement effort.

Some of the problems in measuring improvement are likely to be linked to ongoing controversies about the relevant dimensions of quality and safety and the prioritisation of different types of measures, including, for example, the process versus outcome debate.^8 9 12^ Other problems are likely to be more mundane, relating, for example, to issues in establishing data collection systems.^10^ Importantly, the literature suggests that some problems may also be linked to ownership: studies of measurement have tended to focus on quality measures generated externally to clinical teams (eg, by regulators or payers), which clinicians may not perceive as directly relevant to their everyday concerns. One suggestion is that clinicians may lack engagement because they perceive externally imposed measures as having little or no relevance to their clinical work and as little more than an administrative burden.^8^ Yet little evidence exists on what happens when clinical teams themselves choose their own measures (rather than having to use those selected externally) and design and implement data collection systems that they see as fitted to their own local circumstances. A well-characterised account of responses to such an opportunity would be very useful.

In this article, we use data from a mixed-methods independent evaluation of a nine-site UK patient safety improvement programme to report on clinical teams’ experiences of using locally selected measures. We aimed specifically to describe their experiences of planning and conducting measurement activities, collecting data and analysing and interpreting data for their improvement projects.

## Methods

### The Safer Clinical Systems improvement programme

The study we report is based on data from an independent evaluation of a patient safety improvement programme run in the UK, which used an approach known as Safer Clinical Systems.^7^ Based on methods of risk management and improvement used in other hazardous industries, the Safer Clinical Systems approach seeks to enable organisations to make improvements to local clinical systems and pathways using a structured methodology for identifying risks and for modifying or re-engineering systems to control risk and enhance reliability.^7 13^ It involves a series of steps in which teams define a clinical pathway and its context; do a detailed diagnostic assessment of the pathway to identify risks and hazards; assess and select options for change and develop an action plan to implement them; and undertake system improvement cycles involving implementation, evaluation of progress against a measurement plan and revision of interventions.

Measurement is a key element of Safer Clinical Systems. During the course of the programme, participating teams were expected to: (1) develop a detailed measurement plan to set out outcome and/or process measures that were appropriate for collecting useful data; (2) establish data collection systems; and (3) analyse and interpret their data using Statistical Process Control (SPC) (Box 1).

Box 1 **Measurement in the Safer Clinical Systems approach**

Measurement is a key element of the Safer Clinical Systems approach, which emphasises local ownership and local selection of measures for the monitoring of improvement. The approach does not recommend or impose any external measures, though it does recommend that Statistical Process Control (SPC) be used as a means of monitoring and analysis of data.

SPC is an approach to understanding and acting on variation observed in measured properties of a system. In this approach, data are used to gain insight into how a healthcare system or process is performing, and how this performance is changing over time. These insights inform actions on the system, targeted at causes of poor performance. Continuing analysis is used to understand whether these actions have led to improvement.

Control charts are the main analytical tool used in SPC.^18^ A control chart shows a time series of how the measure varies over time. The centre line represents typical performance of the process or outcome that the team is seeking to improve. Control limits (dotted lines parallel to the centre line) show the degree of variation that is to be expected assuming that the process or outcome being measured has not changed. SPC provides sets of rules that are used to assess a time series for the presence of special cause variation – evidence that performance has changed.

A distinctive feature of Safer Clinical Systems is that it does not try to impose predefined solutions but instead seeks to help organisations develop their own capacity to detect and address weaknesses in their systems and to measure and report their improvement outcomes. It does so by offering training on a range of improvement tools and techniques (including how to measure for improvement) and emphasising the need to engage local staff (clinical and managerial) in improvement attempts.

Funded by an independent charity (The Health Foundation), the programme was run in a total of nine UK hospitals in two phases: the main phase, which ran 2011–2014 and included eight sites, and the extension phase, which ran 2014–2016 and included six sites (five of the original sites plus an additional one that had not taken part in the main phase). Each of the nine hospitals taking part in the programme used the Safer Clinical Systems approach to proactively assess risks and hazards in their clinical pathways and to develop effective risk-control interventions (table 1).

**Table 1:** Sites involved in each phase of the programme and in independent SPC analysis by the evaluation teams

	Aim of the project		Data subject to independent SPC analysis		Key challenges and achievements in measurement	
	Main phase	Extension phase	Main phase	Extension phase	Main phase	Extension phase
Loganberry	Improve the medication management pathway for patients with Parkinson’s disease between primary and secondary care.	Improve the recognition and treatment of venous thromboembolism in patients admitted for surgery.	No (data were not suitable for SPC analysis).	Yes, data from six measures.	Six measures reported in safety set (no data were available on one of these). SPC analysis does not appear to have been used; simple before and after comparisons are reported. The small numbers of patients meeting inclusion criteria made it difficult to analyse for change over time for all measures.	Six measures reported in safety set. The team were able to collect data against measures, but no explicit baseline period was stated. Operational definitions were ambiguous (eg, no meaning ascribed to “Not Applicable” for some variables). Sampling procedure was described as random, but it was not clear from what population this was taken nor how this was done.
Elderberry	Improve prescribing accuracy for patients with an acute medical illness by developing a prescribing pathway between the emergency department and the emergency admissions unit.	Improve the accuracy of prescriptions in the emergency admission unit (follow-up).	No (not included in sample for independent analysis).	Yes, data from seven measures.	Of the five measures in the original safety set, only two were reported on at the end of the project. Although this site was not included in the sample for independent SPC analysis, the measurement report produced by the team indicates that SPC analysis, presentation and interpretation for two measures was conducted appropriately.	Seven measures reported in safety set. No formal measurement plan was developed, but elements of measurement planning appeared in other project documents. Unclear how denominator was established for three of these measures. Sampling procedures were unclear. Despite this, overall data quality was high with little missing or invalid data. The team’s measurement ability seemed to improve over the course of the project.
Beechtree	Improve the handover across primary, secondary and tertiary care for children with complex illnesses.	Improve the recognition of, and timely response to, sepsis or potential sepsis in patients admitted to the emergency department.	No (not included in sample for independent analysis).	Yes, data from four measures.	Seven measures reported in safety set. No data were available on two of these measures. Many missing data on two measures, and data on two further measures were difficult to interpret due to poor quality presentation.	Four measures reported in safety set. No formal measurement plan was developed. Operational definitions were initially unclear but were clarified later in the project. Data quality was poor in places leading to difficulties in interpretation.
Cedartree	Improve safety and effectiveness of the transfer of care between daytime and out-of-hours clinical teams.	Refine the Safety Case approach, explore its scalability and its potential application to a complex pathway (ie, surgery).	Yes, data from five measures.	No (no data were collected).	Five measures reported in safety set. The large amount of data collected by this team placed considerable strain on resources. The team lacked experience to access and use the relevant data and IT systems and initially struggled balancing desired data collection against available staff time, leading to some missing data. Operational definitions of some measures were unclear. Only 1 month of baseline data were available, making it hard to evaluate improvement.	The project stalled after the diagnostic phase. No risk controls were implemented, and no measurement plan was in place.
Rowanberry	Reduce the number of medication-prescribing errors that reached the patient in the acute medical unit.	Improve the reliability and accuracy of medicines reviews and reconciliation on admission and discharge.	Yes, data for four measures.	Yes, data from three measures and 10 submeasures.	Four measures reported in safety set. The measurement plan evolved over time, as the team realised that they were trying to measure too much and not were not measuring the right things. Team were able to collect data successfully for their revised measures.	Three main measures and 10 submeasures reported in the safety set. Initially, the team planned to record only preaggregated total figures, rather than patient-level data. Following formative feedback, this approach was modified to enable patient-level data to be recorded. The team then successfully established a measurement system providing data on their safety set measures on a regular basis.
Ashtree	Improve the quality and safety of shared care of renal patients receiving surgical intervention.	*Did not take part in this phase.*	Yes, data from six measures.		Six measures reported in safety set. Some operational definitions were ambiguous. Due to missing data resulting in small denominators, some measures suffered from low sensitivity. Baseline periods not clearly stated. The team were able to conduct control chart analysis appropriately for some measures.	-
Hollyberry	Reduce medications prescription errors and increase the safety of the prescribing pathway for patients admitted to the acute medicine admissions unit from primary care or the emergency department.	*Did not take part in this phase.*	Yes, data from four measures.	–	Four measures reported in safety set. Some ambiguity in the operational definitions used by the team (eg, it was not clear what was meant by a ‘complete’ or ‘satisfactory’ drug chart). Missing data led to a lack of sensitivity in some measures. However, the team were able to collect a large amount of data to ascertain the outcomes of their improvement interventions.	
Oaktree	Reduce the number of unplanned readmissions to hospital among patients aged 75 years or older.	*Did not take part in this phase.*	No (data not suitable for SPC analysis).	–	Three measures reported in safety set. During the early part of the project, the team discovered that the way readmission rates were being calculated at their organisation had a flaw and had to undertake a data cleaning exercise to resolve this. The methodology used to detect change was never clearly described.	
Blueberry	*Did not take part in this phase.*	Improve the medication management process and explore the possibility to use the Safety Case approach for assurance purposes.	–	No data were collected.		The project stalled after the diagnostic phase. No risk controls were implemented, and no measurement plan was in place.

AHRQ, Agency for Healthcare Research and Quality; SPC, Statistical Process Control.

In the main programme phase, participating sites received training and guidance and were monitored in their progress, by a dedicated programme support team. Support on measurement included approximately 1 day of training on principles of measuring improvement, SPC and use of software for capturing their data and generating charts. In the extension phase, teams were expected to use the Safety Clinical Systems approach on their own, without the support and control that characterised the main phase.

### Programme evaluation

We undertook an independent evaluation of the Safer Clinical Systems programme using a mixed-method design. We combined a qualitative study, which aimed to describe how participating teams experienced taking part in the Safer Clinical Systems programme, with expert review of measurement plans and analysis of data collected for the programme.

#### Qualitative study

We conducted semistructured interviews with members of the participating teams and unstructured ethnographic observations of teams’ activities related to programme participation. Particular effort went into capturing how teams undertook tasks relating to measurement (eg, identified and selected their quality measures and developed and implemented a measurement plan to assess the impact of their improvements). We were also interested in characterising the challenges and hurdles faced by the teams in doing so.

Observations and interviews were conducted by non-clinical researchers who were members of the evaluation team. Interviews were conducted in person or on the phone, were recorded digitally and transcribed verbatim. All interviewees signed an informed consent form. Observations were unstructured and included routine clinical activities, team meetings and informal chats with relevant staff. Extensive field notes were taken during visits, and researchers were then debriefed by other members of the evaluation team on return from visits.

#### Expert review of measurement plans

All the measurement plans prepared by the participating teams were reviewed by one author (TW), who is a specialist in measurement for improvement and an expert in SPC. Published checklists aimed at improving the quality of measurement were used as review criteria.^3 14^ The level of information on each step of the measurement process provided by teams was deemed unclear if it was not sufficient for author TW to know how to repeat the measurement process. The reviews of these plans were used for evaluation purposes (eg, to assess the quality of the plans) and to provide formative feedback to the participating teams. This feedback was provided to each team through ad hoc coaching sessions, led by author TW and the programme support team in the main phase, and author TW alone in the extension phase. Up to two such sessions, conducted by telephone, were offered to each team.

Towards the end of each phase of the programme, TW reviewed and independently analysed the raw data from a selection of the participating sites (four sites in the main phase and four sites in the extension phase). We initially sought to select sites that would ensure the greatest diversity of projects. However, some sites did not produce data or produced data unsuitable for analysis that could not be included. Table 1 illustrated the four sites in each phase that had their raw data subject to independent SPC analysis.

#### Data analysis and synthesis

Analysis of the qualitative data was based on the constant comparative method, inductively generating thematic categories and using the literature on measurement and quality improvement as sensitising concepts.^15^ We first analysed data site by site to ensure that it was understood in terms of relevant context. Then, for each site, we integrated qualitative data and findings from the expert review to produce a comprehensive and in-depth picture of sites’ experiences of measurement. Finally, we conducted cross-site analysis in order to develop higher level concepts and broader learning on measurement.

## Results

Across the two phases of the study (main and extension), the qualitative evaluation study involved 862 hours of observation and 143 interviews (table 2) covering all aspects of the programme (not just measurement). The participating site teams specified, between them, a total of 67 measures that they planned to use to monitor their processes before and after introduction of their risk-control interventions. The data for 49 of these measures—which were sourced from four of the eight sites participating in the main phase and from four of the six sites participating in the extension phase—were independently analysed by the evaluation team (table 2).

**Table 2:** Data collected in the evaluation of each phase of the programme

	Qualitative data	Quantitative data
Main phase	668 hours of observations. 94 recorded interviews.	Measurement plans for eight sites. Data from 37 measures were available for independent SPC analysis, 19 of which were included (data from 18 measures were not amenable to independent analysis).
Extension phase	194 hours of observations. 49 recorded interviews.	Measurement plans for six sites. Data from 30 measures were included in independent SPC analysis.
Total	862 hours of observations. 143 recorded interviews.	Data from 67 measures, 49 of which were included in independent SPC analysis.

SPC, Statistical Process Control.

The clinical teams participating in the programme typically comprised a clinical lead (often a senior physician), a project manager, others from a clinical or managerial background and an executive sponsor (a senior individual who reported to the board but was not involved in day-to-day running of the project). The participating sites varied in the extent to which they enjoyed active support from executive or non-executive board members and from other clinicians; the interaction of the work with infrastructure such as large IT system projects; a pre-existing audit culture and organisational capability for managing complex data; and the resources available to the teams, including release of staff to undertake project work. In the account below, we offer an analysis of measurement-specific issues and specifically on teams’ ability to: (1) manage the tasks associated with developing measurement plans; (2) establish and use reliable data collection systems; and (3) analyse and report data in appropriate ways. Our analysis is focused on drawing out generalisable learning across the programme and does not seek to compare/contrast sites. Table 1 provides a summary of each project’s aim and measures and keychallenges and achievements in measurement.

### Developing a detailed measurement plan

The measurement plans that the teams were asked to develop were intended to identify and define suitable measures in advance of any improvement interventions being implemented and to specify a sampling and analytical strategy. In the main phase (in which participating teams received dedicated measurement support and guidance), all teams produced a measurement plan document; in the extension phase, two out of the six teams did so. When no measurement plan was available, the evaluation team assessed any written material provided by the teams that included elements of measurement planning. Our review of the measurement plans (or related documents) indicated that most demonstrated great enthusiasm and also multiple problems; here, we describe six.

The first problem was the overambitious nature of the plans. Several teams initially identified very many measures (up to 15 in some cases) that were highly diverse in character. Given the formative nature of the evaluation, these sites were asked to reduce the number of measures in their final measurement plans to five or six and to concentrate their efforts on those (table 1 reports the final number of measures used by each team after feedback).

Second, many plans did not demonstrate the level of specification or understanding of the underlying methodological principles necessary to gather good quality data, consistent with varying confidence about measurement expressed in interviews.

I am very confident actually, very confident our data is accurate, given the sort of work we did around some of the reliability and the training. (Interview, main phase of the programme)

The measures are the bit that we're struggling with the most at the moment, using the BaseLine software I'm not finding easy at all, I'm struggling with it…. I don't feel that I've had enough training in it. (Interview, main phase of the programme)

Every measurement plan contained examples of operation definitions of measures that were imprecise, lacked important details or were difficult to understand by those outside the project team. For example, some sites used compliance with a care bundle (eg, medication reconciliation or review) as a measure but did not always specify the operational definition of the individual components of the bundle. One team used a measure labelled as ‘Number of patients […] who have their medicines 100% correct at 24 hours’ without specifying how staff should ascertain that medicines were correct. Similarly, terms such as ‘delay”, ‘error’, ‘time zero’ and ‘baseline’ were not fully defined, leaving room for different interpretations between observers and over time. When sites reported that some data were ‘not applicable’ to certain measures, they did not always give a reason for this.

Different names are used to refer to the same measures in this document when compared with the others, and also in different parts of the same document. For example, the following two measure names seem to be used interchangeably: ‘% of patients on EAU [emergency assessment unit] who have all their medicines correct at 24 hours’ and ‘Accuracy of prescription at 24 hours on EAU’. (Evaluation team’s review of the measurement plan, extension phase)

The third problem was that some measures selected by the teams were insufficiently sensitive to capture the spectrum of improvements sought by the sites. For example, one site’s definition of compliance with its medication reconciliation bundle stated that all 10 elements of care in the bundle should be in place. Even if nine elements of the bundle were in place and one was not, the patient’s care was deemed non-compliant. Bundles should usually include fewer elements (three to five),^16 17^ suggesting perhaps suboptimal design of the bundle and also indicating that full compliance was unrealistic and that use of this measure might fail to detect potential improvements.

Fourth, specification of sampling procedures was typically weak, and it was often unclear what procedure was to be used for random selection. Inclusion and exclusion criteria necessary for determining who or what should be counted were often unclear. For instance, one site in the extension phase reported in their measurement plan that ‘each week a random sample of 5 patient case notes should be selected for admission, transfer and discharge’, without specifying how such random sampling should be done. If, for example, staff selected patients randomly from physical stacks of notes, bias might be introduced if some patients’ notes were unavailable.

The fifth problem was that some selected measures were not logically linked to the improvement actions they implemented. For example, one site opted to measure the average proportion of patients going to the operating theatre with a completed perioperative care plan, but then struggled to implement an intervention that would increase completion of the plans. In this site, due to uncertainty about the renewal of the hospital IT contract, it was difficult to make available documents relevant to surgery on the trust’s IT system at the operation stage, and this improvement action was therefore abandoned. Thus, although the site recorded an improvement between the two measurement periods (from 65% to 78% of patients with a complete plan), it was difficult to attribute the site’s improvement to its Safer Clinical Systems project.

Sixth, in general, the measurement plans produced by the teams did not look sufficiently far ahead. For example, the plans did not contain the specifics of how the data would be analysed, thus impacting on important considerations such as the appropriate length of the baseline and how much data over what period would be needed to establish whether an improvement had been made. Most plans did not touch on who was responsible for taking action for improvement based on the findings of the analysis or on embedding measurement in routine care.

### Collecting data

Interviews and observations showed that teams generally struggled to set up and run data collection systems and that running the systems consumed a huge amount of time and resource at several sites. Some challenges were related to teams’ decisions to use entirely new measures for the first time (including ‘home-grown’ measures). Some teams started by using lengthy, unwieldy manual data collection forms that were sometimes amended or abandoned after a short time. In other cases, teams used routinely collected data, but these data were often not as clean or well set up as originally anticipated and often required extensive effort to bring them up to a standard suitable for use.

It’s been a nightmare actually… We’ve been looking… at readmissions, and in retrospect I don’t think the organisation had a consistent metric for readmissions, in terms of what it meant and how they were collecting it. A lot of people that were being classified as readmissions weren’t being readmitted, a lot of people were double-counted or triple-counted or worse, and of course then we had really untidy data… (Interview, main phase)

I had to write a database with the coders, had to pay… the data people to give us the feed of the patients going to [operating rooms]. [This] took a huge amount of time and it meant that until February I was manually having to get that data from systems which was an absolute nightmare. But it's better now. (Interview, main phase)

Data collection often depended on voluntary, unpaid or extra activity that was unsustainable.

It certainly has been extra work for all of us, for example observing the handover is not something that would normally be part of my day-to-day job. But obviously it has been a time investment. We have used the Health Foundation money to pay for part of it, but there certainly has been extra goodwill from people who collected the data. (Interview, main phase)

Data collection systems were not always run exactly as designed, sometimes resulting in missing data. One team struggled to get reliable data collection at weekends. In one site, a special form that was supposed to be used for data collection was not consistently used, with data instead collected in a notebook or on odd pieces of paper in non-standard formats. In another example, attempts to collect data from doctors at the end of night shifts was met with difficulty, as the physicians were tired and wanted to finish their clinical tasks before going off duty.

A further challenge was that teams did not reliably collect baseline data before they introduced interventions aimed at improvement. In part, this was because once the participating sites became aware of the many (and, in some cases, severe) hazards threatening patient safety in their clinical systems through use of the Safer Clinical Systems diagnostic tools, they were understandably eager to address these hazards quickly. Accordingly, some sites proceeded to implement improvement actions before measurement had started. The consequent absence of a baseline period, while well justified in terms of addressing risk, meant that it was difficult to demonstrate that any improvement was attributable to the programme or indeed that the risk was now well controlled.

### Analysing and interpreting data

The teams’ ability to conduct SPC analysis to present and interpret their data was highly variable. Some sites succeeded in analysing data on their key process measures regularly and producing high-quality reports. For example, one site recorded changes in clerked weekday in-hours and out-of-hours admissions or senior reviews within 4 hours of admission; another site analysed the percentage of patients who had the correct medications at 24 hours after admission. These analyses were undertaken to a high level of competence and accuracy. Other sites, however, struggled with undertaking appropriate analysis of their data.

One reason for these differences lay in the variable mix of skills and experience in participating teams. One team included an academic advisor who had expertise in measurement and experience working with the hospital’s Quality Improvement Directorate. Other sites lacked such a skillset:

It would have been great to have someone with data analysis skills, or someone from our informatics team who was dedicated to (our project). We've used up a lot of our teams' time learning where different bits are kept. (Interview, main phase)

In some cases, appropriate analysis was not possible because of basic weaknesses in the measures or data recording, for example, when the definitions used were so poorly specified that it was not clear exactly what was being measured. For instance, in measuring the time from admission to the diagnostic test being done, one team recorded the time but not the date of the test. It was therefore impossible to distinguish whether the test had taken, for example, 4 or 28 hours.

Analysis was also severely hindered by missing data. For example, in one site, missing data for 1 week meant that it was not possible to complete SPC analysis of one of their key measures. This site’s measurement planning did not include any strategies for minimising or mitigating missing data, a common theme across the sites. In another site, data collection was frustrated by the small number of patients meeting the eligibility criteria, complicating any attempts to make valid inferences or draw conclusions about the impact of the intervention. Since very few measurement plans included an advance plan for the type of analysis, many sites failed to exploit some available support tools that would have proved highly beneficial. For example, when using a p-chart,^18^ tables could be used to identify an appropriate frequency of data collection and a subgrouping strategy (ie, weekly percentages vs monthly or other period) to make sure that the chart is sensitive enough to be useful.^19^

Analysis and interpretation were sometimes flawed: we saw evidence of sites assuming that their data provided evidence of improvement, but such interpretations were not always backed up by sound statistical analysis. One site reported improvement on a measure from around 75% to just under 90%, but no statistical analysis was presented to back up this claim. Visual inspection of a time series chart seemed to show a potentially unstable process; it was therefore impossible to draw accurate conclusions from such statements. Another site presented data for two measures that showed possible evidence of improvement, but without SPC analysis, it was not possible to conclude whether this was true improvement in the process.

## Discussion

Our study suggests that improvement programmes that emphasise local ownership and local selection of measures, such as Safer Clinical Systems, may not escape the pervasive challenges of high-quality measurement. Teams may struggle to produce a high-quality measurement plan, to provide clear definitions of their measures and data or to complete data collection and analysis reliably. Participating teams often selected unreliable or inappropriate measures, some of them ‘home-grown’, indicating that an emphasis on local ownership of measures is not always compatible with the need to use the kind of validated measures that are most suited to producing sound, credible evidence. The range of skills required to collect and analyse data was not generally sufficient in most teams, despite some training being provided by the programme. These challenges were pervasive across sites irrespective of the specific data, measures and analytical approach adopted, to the point that they may indicate a systemic problem.^7^

We did not aim to explain differences across sites, nor to identify the contextual factors promoting or inhibiting effective measurement in each setting, but our analysis does allow insight into widespread challenges that may hamper teams’ measurement efforts. The effective execution of measurement across the lifespan of a quality improvement initiative requires expertise in a range of technical activities: selecting measures, high fidelity data collection, statistical analysis and interpretation of results. The consequences of decisions made in the early stages of designing measures may not become apparent until the middle or end of an improvement initiative, by which time it may be too late to secure high-quality data. Experience of working on several improvement initiatives provides valuable insights into the constraints of data access and collection in healthcare systems and enables more realistic planning for future initiatives. This study highlights the tendency for teams without such knowledge and experience to underestimate the challenges they may face in enacting their measurement plans and the resulting mismatch in skills and time dedicated to measurement activities. All of this indicates the need for clinical teams to access dedicated capability and capacity for measurement. The optimal approach to building or sourcing this capability and capacity across local and national health systems is not clear, and it seems likely this will take considerable time to achieve.^20^ Our study does suggest that it is not realistic to assume that brief training interventions and manuals will be enough to bring most clinical teams fully up to speed.

As well as measurement-specific issues, broader characteristics of the organisational setting, well established in the literature as influencers of success in improvement, played a role in enabling or hindering the teams’ ability to measure effectively.^21–23^ This complexity means that narrow interventions are unlikely to be sufficient to improve the quality of measurement in quality improvement. Some relatively simple forms of support might, however, be helpful. Existing repositories of validated measures, such as the US-based Agency for Healthcare Research and Quality (AHRQ) Measures Clearing House, are important resources that may not be having their desired impact; teams in this study did not reference such repositories in their plans. This may be due to a lack of awareness that such repositories exist, limitations in the coverage and relevance of repositories for specific improvement initiatives and deficits in technical skills and knowledge needed to use centrally constructed measure definitions. Further research is needed to understand these issues and to support healthcare professionals to capitalise on existing resources.

Improving access, and ability to use, repositories of validated measures could go some way to addressing some of the challenges we identify. Increasing transparency in reporting (eg, publishing failed measurement attempts and inappropriate endpoints) might serve the same purpose while enhancing the evidence base for measurement. Yet, such actions are likely to have limited impact without a broader programme of measurement capability building at a system level. The definitions of measures will always need to be interpreted and implemented in local systems to account for variation in processes across settings.^10^ Increased capability in measurement is necessary for staff at all levels to understand and act in response to measures.

Our study of measurement does have limitations. Although we used established techniques (eg, integrating multiple sources of data and collecting data at multiple points in time) to improve the rigour and generalisability of our analysis, our study is limited by the fact that we have examined a specific improvement programme, which took place in hospitals only and was focused on the identification of risks in clinical systems.

The potential benefits of robust measurement in improvement initiatives are clear. Yet it is not clear how best to realise those benefits in practice. Countering the problems of measurement that hinder quality improvement projects is vital, not least because they undermine the credibility of the enterprise and diminish the prospect that clinicians will take the results seriously in future improvement efforts. Unfortunately, using a local ownership approach does not seem to provide an escape from these problems. We conclude that, in driving improvement, the data (and the data source) need to be seen as credible by potential participants, while at the same time not too irksome or burdensome to collect,^8^ but how to achieve an optimal balance between expert input and local ownership requires further examination. Future research should focus on understanding how measurement can be planned, executed and drawn on to best support improvement in quality of care for patients. Different models for measurement, aiming to address the challenges highlighted in this study, should be evaluated. Such models could include those that involve more extensive training of NHS personnel, loaning of information analysts to improvement teams, resources to support better planning and execution of measurement and provision of consultancy expertise.
